# Requirement for STAT3 and its target, TFCP2L1, in self-renewal of naïve pluripotent stem cells *in vivo* and *in vitro*

**DOI:** 10.1242/bio.059650

**Published:** 2023-01-17

**Authors:** Sophie Kraunsoe, Takuya Azami, Yihan Pei, Graziano Martello, Kenneth Jones, Thorsten Boroviak, Jennifer Nichols

**Affiliations:** ^1^ Wellcome Trust – Medical Research Council Stem Cell Institute, University of Cambridge, Jeffrey Cheah Biomedical Centre, Puddicombe Way, Cambridge CB2 0AW, UK; ^2^ Department of Physiology, Development and Neuroscience, University of Cambridge, Tennis Court Road, Cambridge CB2 3EG, UK; ^3^ Department of Biology, University of Padua, Padova 35121, Italy; ^4^ Centre for Trophoblast Research, University of Cambridge, Cambridge, UK

**Keywords:** Naïve pluripotency, Embryonic diapause, STAT3, TFCP2L1, Blastocyst, Embryonic stem cells

## Abstract

We previously demonstrated gradual loss of epiblast during diapause in embryos lacking components of the LIF/IL6 receptor. Here, we explore the requirement for the downstream signalling transducer andactivator of transcription STAT3 and its target, TFCP2L1, in maintenance of naïve pluripotency. Unlike conventional markers, such as NANOG, which remains high in epiblast until implantation, both STAT3 and TFCP2L1 proteins decline during blastocyst expansion, but intensify in the embryonic region after induction of diapause, as observed visually and confirmed using our image-analysis pipeline, consistent with our previous transcriptional expression data. Embryos lacking STAT3 or TFCP2L1 underwent catastrophic loss of most of the inner cell mass during the first few days of diapause, indicating involvement of signals in addition to LIF/IL6 for sustaining naïve pluripotency *in vivo*. By blocking MEK/ERK signalling from the morula stage, we could derive embryonic stem cells with high efficiency from STAT3 null embryos, but not those lacking TFCP2L1, suggesting a hitherto unknown additional role for this essential STAT3 target in transition from embryo to embryonic stem cells *in vitro*.

This article has an associated First Person interview with the first author of the paper.

## Teaser

**Summary:** Inducing diapause in mouse embryos demonstrates that STAT3 and TFCP2L1 are essential for self-renewal of the epiblast, but only TFCP2L1 is also required for derivation of embryonic stem cells.

## INTRODUCTION

Embryonic stem cell (ESC) lines derived from epiblasts of preimplantation mouse embryos (Evans and Kaufman, 1981; Martin, 1981) have been used extensively to study and model mammalian development, since they can be expanded in culture, whilst retaining the ability to differentiate into all tissues of the body. This flexible state is known as ‘naïve pluripotency’ (Nichols and Smith, 2009). ESCs can self-renew in medium supplemented with leukaemia inhibitory factor (LIF) (Smith et al., 1988; Williams et al., 1988), operating via signal transducer and activator of transcription (STAT)3 (Burdon et al., 1999; Matsuda et al., 1999; Niwa et al., 1998). The LIF receptor complex comprising LIFR and gp130 (also known as IL6ST) activates Janus-associated kinases (JAKs), which phosphorylate STAT3 at tyrosine 705 (pY705) (Ni et al., 2004; Zhang et al., 2000). Mutation of pY705 ablates ESC self-renewal in standard (serum/LIF) culture conditions (Huang et al., 2014); LIF or related cytokines were therefore previously considered essential for ESC self-renewal. However, a refined serum-free culture regime, ‘2i’, based upon dual inhibition of glycogen synthase kinase (GSK)3 and MEK/ERK, allows ESC derivation from STAT3 mutant embryos by incubation from the morula stage (Ying et al., 2008). STAT3 null ESCs enabled analysis of downstream signalling events associated with ESC self-renewal, and thus characterisation of the signalling network operative during maintenance of naïve pluripotency *in vitro* (Martello et al., 2013; Ye et al., 2013). The most significant player emerging from this analysis was TFCP2L1 (also known as CRTR-1), the forced expression of which could rescue STAT3 null ESCs in serum/LIF culture. Using pathway analysis and computational modelling, an essential role for TFCP2L1 in ESC maintenance was proposed and supported experimentally (Dunn et al., 2014). Moreover, transfection of *Tfcp2l1* into epiblast stem cells derived from postimplantation epiblasts (Brons et al., 2007; Tesar et al., 2007) could direct reprogramming from primed to naïve pluripotency, confirming participation of TFCP2L1 in the naïve pluripotency network (Martello et al., 2013; Ye et al., 2013).

Combined maternal and zygotic deletion revealed an essential requirement for STAT3 during blastocyst expansion, confirming its suspected function in epiblast formation (Do et al., 2013). However, perdurance of maternal STAT3 protein in zygotic null embryos permits developmental progression beyond cleavage stages, allowing them to implant in the uterus, where they acquire abnormalities (Takeda et al., 1997). Interestingly, mutation of either LIFR or gp130 results in considerably less severe phenotypes (Li et al., 1995; Yoshida et al., 1996), supporting previous suggestions of additional alternative requirement for STAT3 signalling in early mouse development (Kristensen et al., 2005).

In normal laboratory rodents, the state of naïve pluripotency is relatively transient, lasting no more than 2 days. It is therefore debatable whether self-renewal of naïve pluripotent stem cells occurs at this stage during uninterrupted development. Conveniently, murine preimplantation embryogenesis can be prolonged by diapause, a natural, facultative phenomenon ensuing when a dam conceives whilst suckling a previous litter. Embryos progress to the peri-implantation stage, embryonic day (E)4.5, but delay implantation until a source of oestrogen is regained. Diapause can be achieved experimentally by ovariectomy prior to the physiological burst of oestrogen secretion at E2.5 (Weitlauf and Greenwald, 1968). Healthy blastocysts are able to sustain diapause for more than a month, then resume normal development (Arena et al., 2021). In previous studies, we showed that epiblast in diapause embryos lacking LIFR or gp130 was gradually reduced by apoptosis, leaving only trophectoderm and primitive endoderm (PrE) (Nichols et al., 2001). Loss of STAT3 or its target, TFCP2L1, may be anticipated to result in a more dramatic phenotype. Here, we show that both STAT3 and TFCP2L1 are critically required for diapause, with almost complete loss of both epiblast and PrE comprising the inner cell mass (ICM) occurring within 4 days of diapause onset (6 days after ovariectomy). Although both pY705 STAT3 and TFCP2L1 are downregulated in the epiblast before implantation, their levels intensify during diapause, consistent with previously published RNA profiling (Boroviak et al., 2015). To enhance optical compartmentalisation of tissue types in peri-implantation and diapause embryos, we developed an image-analysis pipeline to quantify confocal readout of protein levels and distribution for pY705 STAT3 and TFCP2L1. Despite the rapid loss of epiblast in diapaused mutant embryos, we show that capture of self-renewing ESCs from STAT3 null embryos in culture is as efficient as from wild-type (WT) and heterozygous embryos. In contrast, no ESCs could be derived from embryos lacking TFCP2L1, indicating alternative functions for this factor in replication of naïve pluripotent epiblast cells that is not directed by STAT3 signalling.

## RESULTS

### STAT3 pY705 and TFCP2L1 peak in epiblast during embryonic diapause

**Fig. 1.**
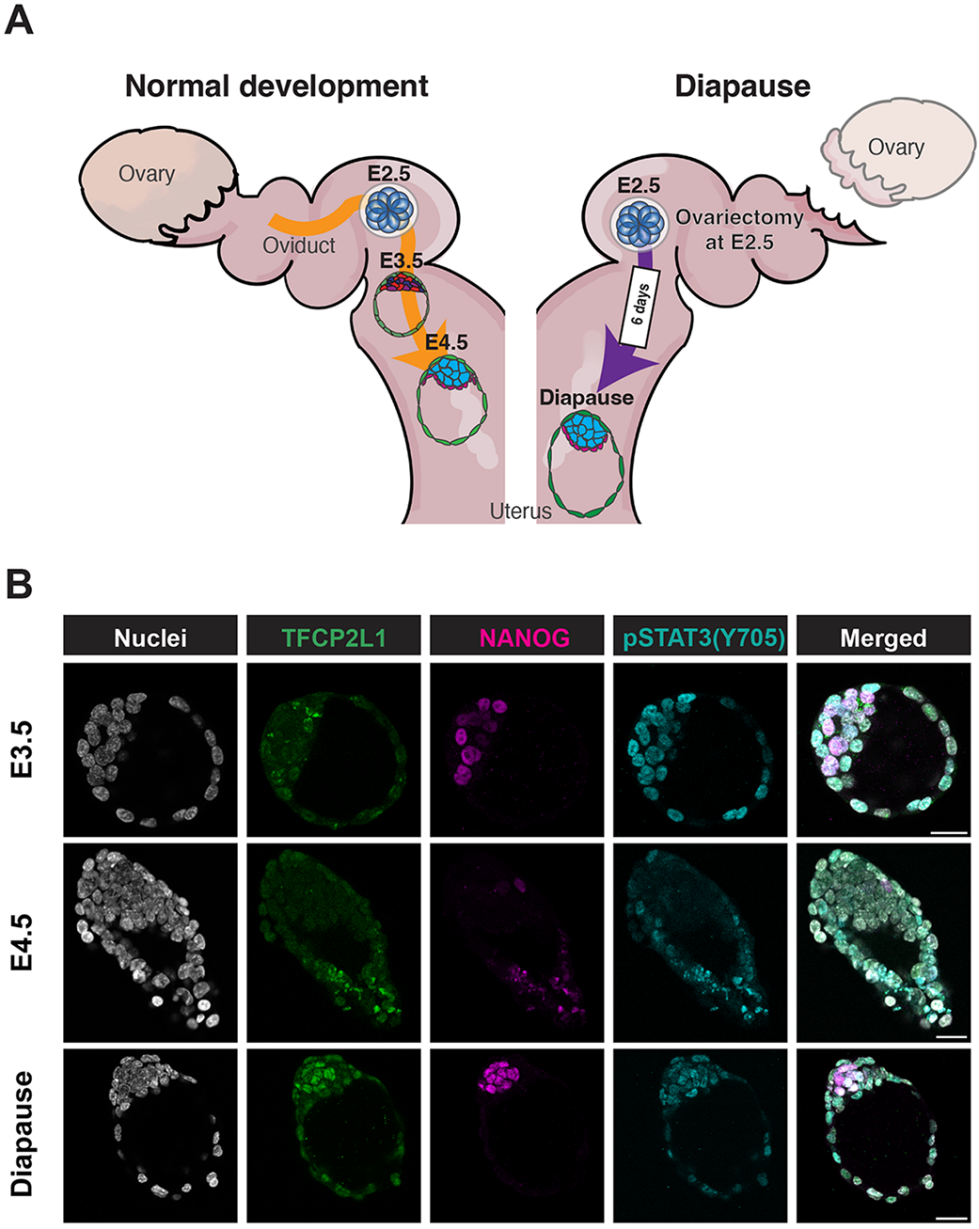
**Distribution of pSTAT3(Y705) and TFCP2L1 in preimplantation and diapause embryos.** (A) Schematic of the mouse reproductive system, representing normal preimplantation development (left) and induction of diapause (right). Diapause was induced by surgical removal of both ovaries at E2.5 (before the physiological burst of oestrogen production), and embryos were collected 6 days later, following migration from oviduct to uterus, thus being diapaused for 4 days. (B) Confocal images of TFCP2L1, NANOG and pSTAT3(Y705) IF in preimplantation E3.5, E4.5 and diapause embryos. Scale bars: 20 µm.

The potential roles of STAT3 and TFCP2L1 in sustaining pluripotency *in vivo* were investigated via induction of diapause (Fig. 1A), as described previously (Nichols et al., 2001). Bilateral ovariectomy was performed on WT female CD1 mice 2.5 days after mating by males of the same strain. Embryos were flushed 6 days later, and were therefore in the state of diapause for 4 days. Immunofluorescence (IF) using antibodies raised against NANOG, STAT3 (pY705) and TFCP2L1 was performed using our standard protocol (Silva et al., 2009), but with methanol permeabilisation, on non-diapaused embryos at E3.5 and E4.5 and following 4 days of diapause (Fig. 1B).

**Fig. 2.**
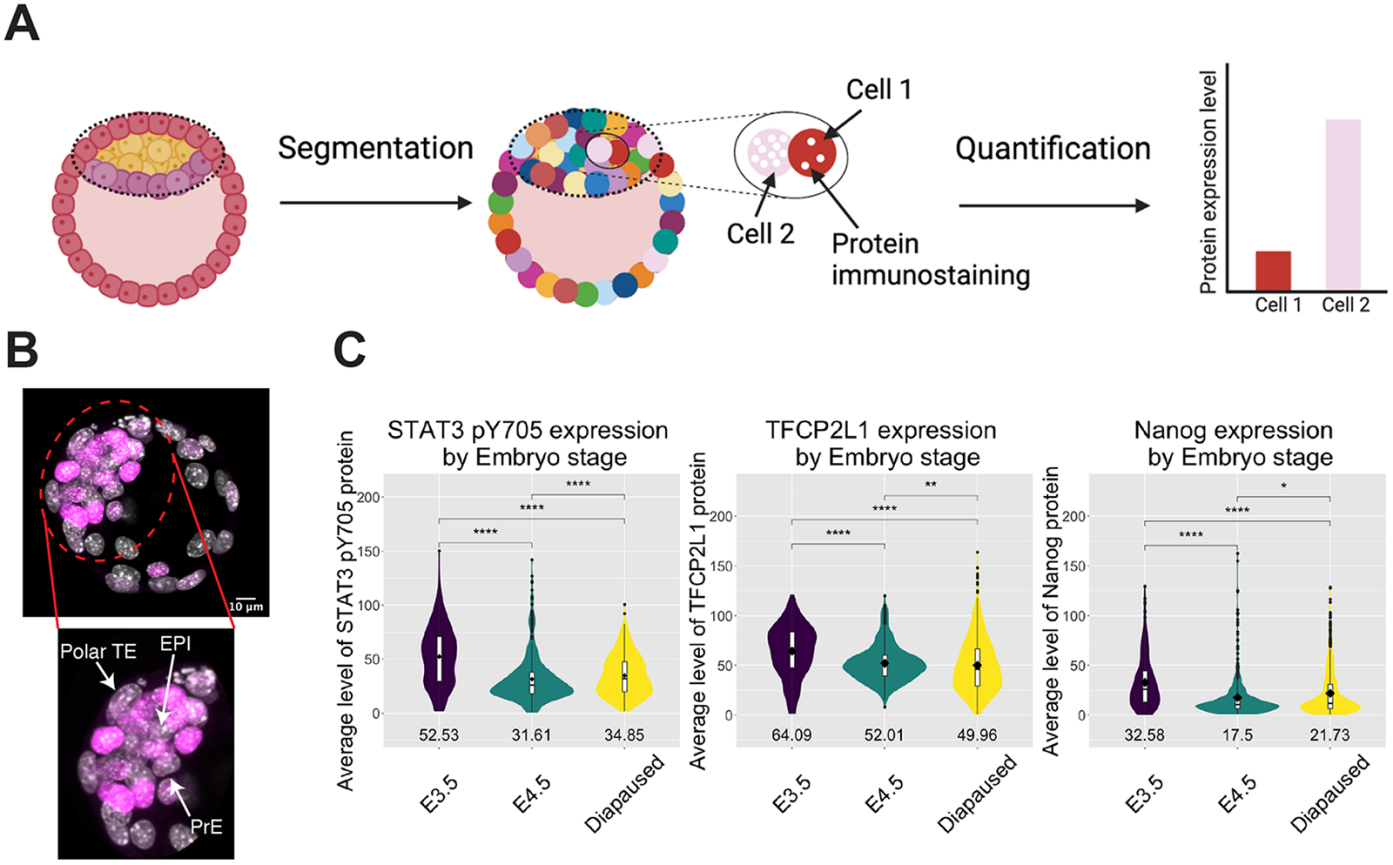
**Development of analysis pipeline for preimplantation, peri-implantation and diapaused mouse embryos.** (A) Schematic of image-analysis pipeline to segment individual nuclei of the embryonic region in 3D and quantify fluorescence of each channel. The region of each embryo to be quantified is depicted by a dotted line oval, thus excluding most of the mural trophectoderm region that does not participate in subsequent formation of the foetus. (B) Top: confocal image of E3.5 blastocyst (grey, DAPI; magenta, NANOG). Bottom: cropped image used in the analysis pipeline with each lineage labelled, showing that the majority, but not all, of the mural TE was removed, whereas the polar TE and hypoblast are not excluded. (C) Violin plots with overlaid boxplots of the integrated density of pY705 and TFCP2L1 expression across each nucleus from each embryo by stage (E3.5, *n*=33; E4.5, *n*=29; diapause, *n*=52). Segmented nuclei were filtered for DAPI signal and volume to remove any erroneous segmentation. Kruskal–Wallis analysis revealed a significant difference in the distribution of STAT3 pY705, TFCP2L1 and NANOG protein between E3.5, E4.5 and diapaused embryos (*P*=3.94E-57, *P*=2.45E-23 and *P*=6.16E-21, respectively). The statistical thresholds indicated in the figure correspond to ns, *P*>0.05; *P≤0.05, **P≤0.01, ***P≤0.001, ****P≤0.0001.

**Fig. 3.**
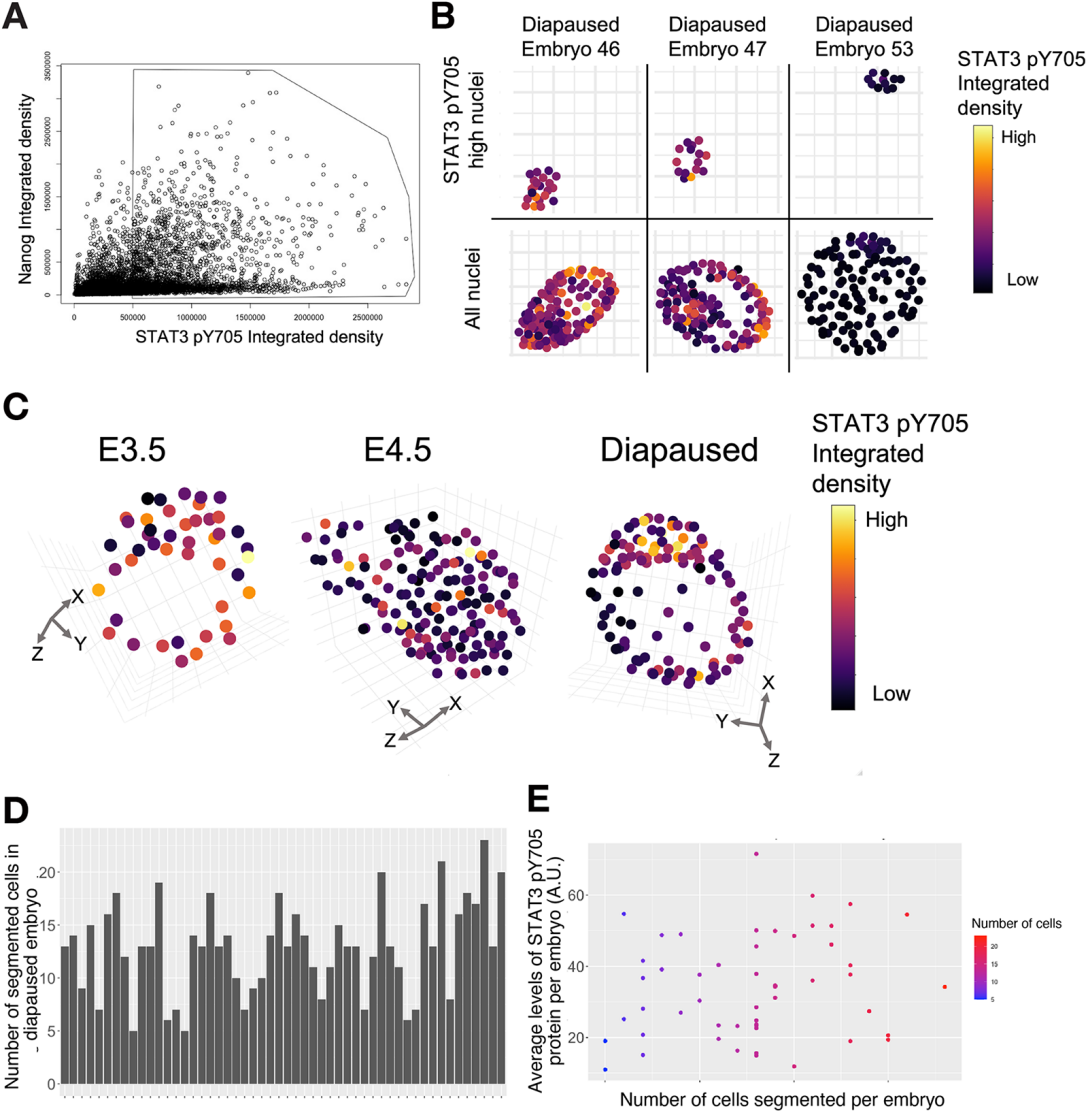
**Example of analysis of embryo IF.** (A) Nuclei are intensity coded by stage dispersed in a 3D co-expression space based on integrated density (sum across all pixel values in the 3D object) of NANOG and pY705 and in each segmented nucleus. The polygon selection encloses cells with a pY705 raw integrated density above 5×10^5^. (B) Distribution of the selected high pY705 cells within the polygon from A in their embryo of origin for three representative diapaused embryos (top) with all nuclei shown below. (C) Representative example embryos from each developmental stage, with nuclei represented by a scatter point and colour coded based on pY705 integrated density. (D) Histogram showing number of cells segmented for each of the 52 diapaused embryos. (E) Dot plot showing the average level of pY705 per embryo, with the number of cells segmented per embryo represented by colour progression from blue (low) to red (high). A.U., arbitrary units.

Quantification for levels of NANOG, pY705 and TFCP2L1 proteins in the embryonic region was enabled by manual cropping to exclude the abembryonic, mural trophectoderm region (Fig. 2A), and application of an image-analysis pipeline assembled from recently developed tools. Fig. 2B is an example of an E3.5 blastocyst with the whole image [4′,6-diamidino-2-phenylindole (DAPI) in grey and NANOG in magenta] and the corresponding cropped image used in the image-analysis pipeline with all lineages labelled. As can be seen in the cropped image, the majority, but not all, of the mural trophectoderm (TE) was excluded; however, the polar TE and hypoblast are not removed. Unfortunately, because *Nanog* is temporarily lost from the epiblast as the embryo implants at E4.5 (Chambers et al., 2003), it cannot be used reliably to define epiblast cells in the peri-implantation embryos. In concordance with visual appearance of the confocal images, a significant increase in both pY705 and TFCP2L1 IF was quantified in diapause embryonic regions compared with non-diapaused peri-implantation embryos at E4.5 (Fig. 2C), consistent with mRNA levels and the transcriptional resemblance of diapause epiblast to self-renewing ESCs *in vitro* (Boroviak et al., 2015). During manual cropping of the images analysed, most mural trophectoderm was removed to prevent these cells confounding the analysis. However, since expression of the proteins analysed is reduced or absent from the remaining TE, distribution of the proteins of interest appears to be bimodal (Fig. 2C). By creating spatially reconstructed *in silico* embryos, it can be observed that cells selected for high levels of pY705 or TFCP2L1 are enriched in the embryonic compartment of diapaused embryos (Fig. 3A,B). In contrast, E4.5 embryos have lower levels of pY705 in their embryonic region compared to E3.5 and diapaused embryos (Fig. 3C). The average level of pY705 for each of the 52 WT diapause embryos appears to vary (Fig. 3D), but does not correlate with the size of the embryonic region (Fig. 3E).

### Propagation of naïve pluripotency *in vivo* requires STAT3 and TFCP2L1

**Fig. 4.**
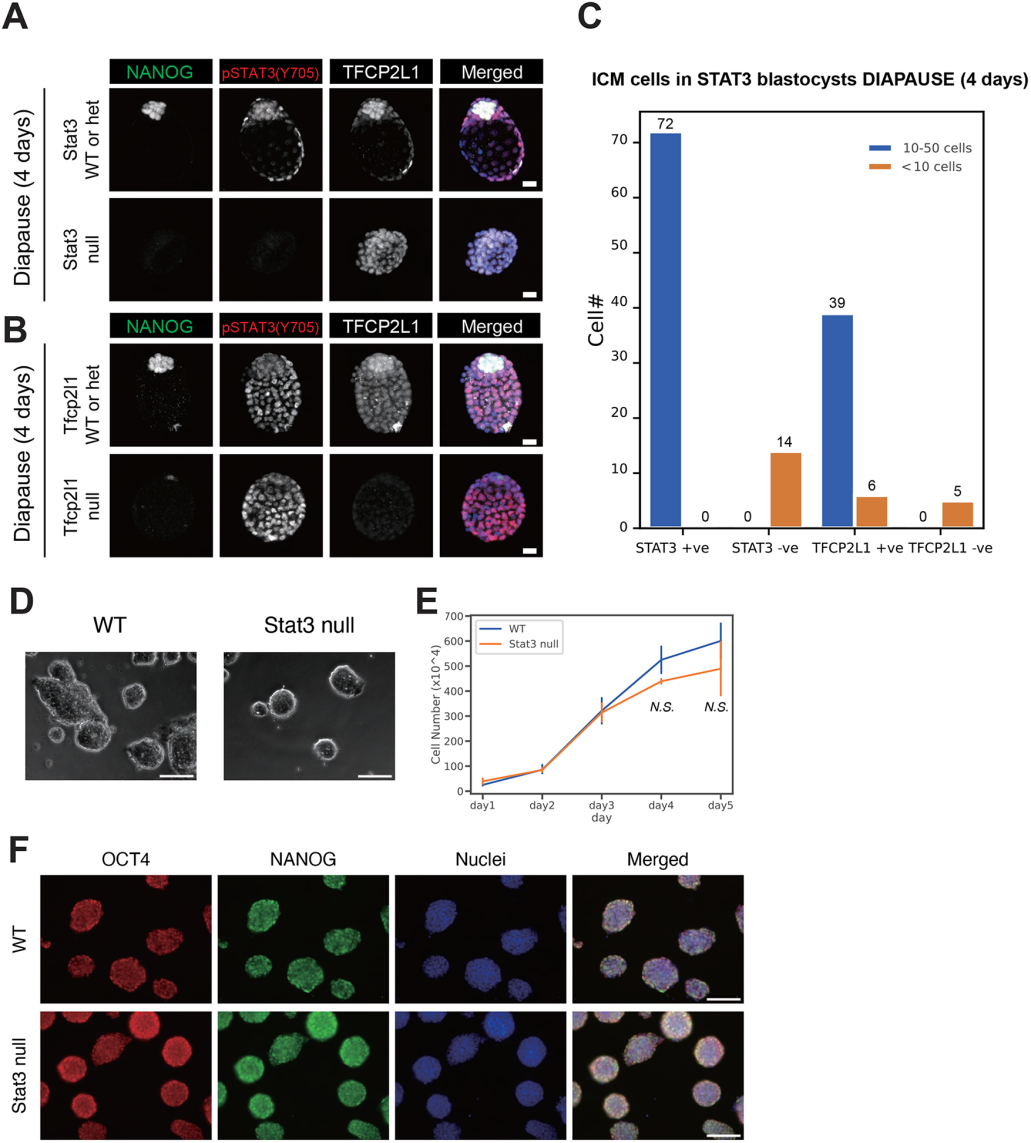
**STAT3 and TFCP2L1 are required for ICM maintenance during diapause, but STAT3 is not required for ESC derivation or self-renewal.** (A,B) IF of NANOG, pY705 and TFCP2L1 in diapause for *Stat3* WT/het and null embryos (A) and *Tfcp2l1* WT/het and null embryos (B). Scale bars: 20 µm. (C) Number of ICM cells in *Stat3* or *Tfcp2l1* WT/Het and null diapause embryos. (D) Brightfield images of WT and *Stat3* null ESCs. Scale bars: 100 µm. (E) Proliferation based on cell number counts of WT and *Stat3* null ESCs cultured in N2B27+2i medium for 5 days (day 4, *P*=0.14; day 5, *P*=0.55 by Student's *t*-test). (F) IF of OCT4 (red) and NANOG (green) in WT and *Stat3* null ESCs. Scale bars: 100 µm.

To assess functionality of STAT3 signalling during maintenance of naïve pluripotency, heterozygous mice were mated *inter se* to generate WT, heterozygous (het) and mutant (null) embryos for *Stat3* or *Tfcp2l1*. Diapause embryos were recovered 6 days after ovariectomy, and IF for NANOG, pY705 and TFCP2L1 was performed. Whereas WT and het embryos possessed large ICMs with many NANOG-, pY705- and TFCP2L1-positive cells, embryos lacking STAT3 or TFCP2L1 exhibited complete loss or severe reduction of the whole ICM (Fig. 4A,B). In both cases, null embryos were under-represented (Fig. 4C), probably attributed to loss before retrieval owing to extreme reduction of the ICM impacting on trophectoderm expansion and embryo integrity. Taken together, these results indicate immediate requirement for STAT3 signalling and functional TFCP2L1 during diapause.

### Capture of ESCs from embryos

Our previous derivation protocol based upon blocking MEK/ERK and GSK3 from the morula stage of development (Ying et al., 2008) was used to capture CD1 background ESCs. Forty-one WT or het and 13 null ESC lines were generated from 56 embryos by *inter se* mating of *Stat3* het mice (Table 1). Each embryo was genotyped by PCR using trophectoderm lysed during immunosurgery (Nichols et al., 1998; Solter and Knowles, 1975). *Stat3* null lines were confirmed by genotyping after expansion. *Stat3* null and WT cell lines could be propagated indistinguishably in 2i medium (Fig. 4D), and no significant difference in cell cycle kinetics could be perceived between them (Fig. 4E). IF for OCT4 and NANOG confirmed naïve pluripotent identity for both WT and STAT3 null ESC lines (Fig. 4F). Conversely, from 65 morulae generated from *Tfcp2l1* het inter-cross, six WT and 49 het, but no null ESC, lines were derived (Table 1), suggesting a distinct requirement for TFCP2L1 in capture of pluripotency *in vitro*.

**Table 1.**
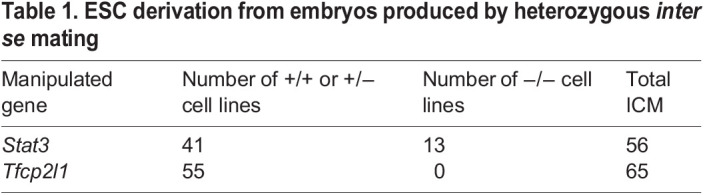
ESC derivation from embryos produced by heterozygous *inter se* mating

## DISCUSSION

Derivation of *Stat3* null ESCs previously facilitated interrogation of STAT3 targets and highlighted TFCP2L1 as the most significant *in vitro* (Martello et al., 2013; Ying et al., 2008). To investigate the potential role of STAT3 and TFCP2L1 in maintenance of naïve pluripotency *in vivo*, we induced embryonic diapause. IF for STAT3 pY705, the protein product utilised for self-renewal of ESCs (Huang et al., 2014), and TFCP2L1 both became visibly enriched in the epiblast during diapause (Fig. 1). This observation was validated using a pipeline specifically developed for quantification of IF images of tightly compacted nuclei (Figs 2 and 3), thus implying a possible role for these factors in epiblast self-renewal *in vivo*. During diapause, in contrast to the phenotype observed following deletion of LIFR or its co-receptor, gp130, which resulted in gradual loss of epiblast, but not PrE (Nichols et al., 2001), embryos lacking either STAT3 or TFCP2L1 lost virtually the entire ICM within only a few days (Fig. 4). This more dramatic phenotype may be a consequence of the precipitous depletion of epiblast, the source of PrE-inducing FGF4 (Yamanaka et al., 2010), compared with deletion of LIF receptor complex components (Nichols et al., 2001). STAT3 also promotes anti-apoptotic activity (Hirano et al., 2000), which could contribute to the enhanced PrE population reported in blastocysts supplemented with IL6 (Anderson et al., 2017; Morgani and Brickman, 2015). We conclude that STAT3 signalling is essential to maintain naïve pluripotency *in vivo* and operates as a signal transducer for pathways in addition to that induced by IL6 family cytokines.

Interestingly, we recently found precocious expression of PrE markers, such as *Pdgfra*, *Sox17* and *Gata4*, in *Stat3* null embryos at the mid blastocyst stage (E3.5), whereas emerging epiblast cells at E3.75 prematurely activated the postimplantation epiblast genes *Utf1*, *Otx2* and its targets *Dnmt3a* and *Dnmt3b* (Betto et al., 2021), which presumably instigated reduction of FGF4 secretion. However, previous observations of PrE persistence during diapause when LIFR or gp130 are deleted (Nichols et al., 2001) imply independence of this branch of STAT3 signalling for PrE maintenance. The present data implicate STAT3 signalling, via TFCP2L1, in PrE maintenance *in vivo*. Our failure to derive ESCs from *Tfcp2l1* null embryos using the strategy that proved highly successful for generation of *Stat3* null ESCs (Table 1) implies an unexpected STAT3-independent role for TFCP2L1 in transition towards *in vitro* self-renewal of pluripotent stem cells. TFCP2L1 plays a role in upregulation of *Nanog* (Ye et al., 2013), which is also indispensable for derivation of ESCs from mouse embryos (Silva et al., 2009). Interestingly, both *Nanog* and *Tfcp2l1* can be deleted from established ESCs cultured in 2i/LIF (Chambers et al., 2007; Martello et al., 2013; Yan et al., 2021), indicating compensation by the robust and redundant network of pluripotency factors that can be assembled in ESCs *in vitro* (Dunn et al., 2014). This pathway connection may explain the failure of embryos lacking TFCP2L1 to yield ESCs and further highlights the distinct requirements for self-renewal of naïve pluripotent cells *in vivo* compared with established cell lines *in vitro*.

## MATERIALS AND METHODS

### Mice, husbandry and embryos

Experiments were performed in accordance with EU guidelines for the care and use of laboratory animals and under the authority of appropriate UK governmental legislation. Use of animals in this project was approved by the Animal Welfare and Ethical Review Body for the University of Cambridge, and relevant Home Office licences are in place.

Mice were maintained on a lighting regime of 12:12 h light:dark with food and water supplied *ad libitum*. *Stat3* mice heterozygous for replacement of exons 20-22 with Neomycin resistance (Takeda et al., 1997) were backcrossed to CD1 mice. *Tfcp2l1* heterozygous mice were generated from ESCs targeted using CRISPR strategy in E14 ESCs obtained from The Jackson Laboratory Knockout Mouse Project (KOMP), via injection into C57BL/6 blastocysts to generate chimaeras. Male chimaeras were mated with CD1 females; grey pups were genotyped by PCR of ear biopsies, and robust males were selected for further backcrossing to CD1 females. Both STAT3 and TFCP2L1 mouse lines were maintained by backcrossing to CD1. Embryos were generated from *Stat3*^+/−^ or *Tfcp2l1^+/−^ inter se* natural mating. Detection of a copulation plug in the morning after mating indicated E0.5. Embryos were isolated in M2 medium (Sigma-Aldrich).

### Genotyping

Mice were genotyped by PCR using ear biopsies collected within 4 weeks of birth, and genomic DNA was extracted using an Extract-N-Amp tissue prep kit (Sigma-Aldrich). Embryos were genotyped using either immunoreactivity to antibody raised against either STAT3 pY705 or TFCP2L1 in the case of those imaged for confocal analysis, or PCR analysis of trophectoderm lysate for ESC derivation. Trophectoderm lysates were each put into 10 µl of lysis buffer [50 mM KCL, 10 mM Tris-HCl (pH8.3), 2.5 mM MgCl_2_, 0.1 mg/ml gelatin, 0.45% NP40, 0.45% Tween 20] added with 200 µg/ml Proteinase K (Thermo Fisher Scientific). The mixture was incubated at 55°C for 60 min, then subsequently heated to 95°C for 10 min. Amplification was carried out with ReadyMix Taq PCR (Sigma-Aldrich) using 5 µl of lysate for 35 cycles (following 95°C hot start for 10 min) of 94°C, 15 s; 60°C, 12 s; 72°C, 60 s, with a final extension at 72°C for 10 min. Reaction products were resolved by agarose gel electrophoresis. Primers used for genotyping PCR are listed in Table 2.

**Table 2.**
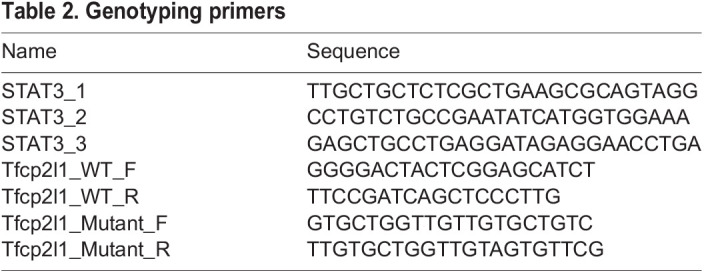
Genotyping primers

### Induction of diapause

To determine the requirement for STAT3 or TFCP2L1 during maintenance of the embryo during delayed implantation, CD1 females or het females mated by het males were surgically ovariectomised under general anaesthesia as described previously (Nichols et al., 2001) before the embryos reached E2.5. Diapause embryos were flushed 6 days later and fixed for IF.

### Derivation and culture of ESC lines

Morulae were collected from het females 2.5 days after mating by het males and used for ESC derivation as described previously (Ying et al., 2008) by culture to the blastocyst stage in KSOM supplemented with 2i, consisting of 1 µM PD0325901 and 3 µM CHIR99021, transfer of ICMs isolated by immunosurgery (Solter and Knowles, 1975) to 48-well plates containing 2i in N2B27 medium, one per well. WT and *Stat3* null ESCs were expanded and maintained in N2B27 supplemented with 2i or 2i/LIF on gelatin-coated plates at 37°C in 7% CO_2_ and passaged by enzymatic disaggregation every 2-3 days. All cell lines were derived and propagated in the absence of antibiotics. No contamination was ever encountered. All cell lines are freely available on request.

### IF

Embryos were fixed in 4% paraformaldehyde for 30 min at room temperature (RT), followed by washing in 0.5% polyvinylpyrrollidone (Sigma-Aldrich) in PBS. Embryos were permeabilised in 0.5% Triton X-100 in PBS for 15 min and blocked with 2% donkey serum, 2.5% bovine serum albumin and 0.1% Tween 20 in PBS (blocking solution) for 1 h at RT. For phosphorylated-STAT3 staining, permeabilisation was performed in absolute methanol for 10 min at −20°C. Primary antibodies were diluted in blocking solution, and embryos were incubated in these overnight at 4°C. After washing in 0.1% Tween 20 in PBS, embryos were incubated with Alexa Fluor-conjugated secondary antibodies at 1:500 dilution in blocking solution in the dark for 1 h at RT. Nuclear staining was carried out with DAPI (Thermo Fisher Scientific) added to the blocking solution for 3×15 min rinses after secondary antibody staining. Embryos were taken through a series of 25, 50, 75 and 100% VECTASHIELD (Vector Laboratories) in blocking solution, then mounted in small drops of 100% VECTASHIELD on glass slides, and coverslips were sealed with nail varnish. Slides were stored at −20°C. Primary and secondary antibodies used are listed in Table 3.

**Table 3.**
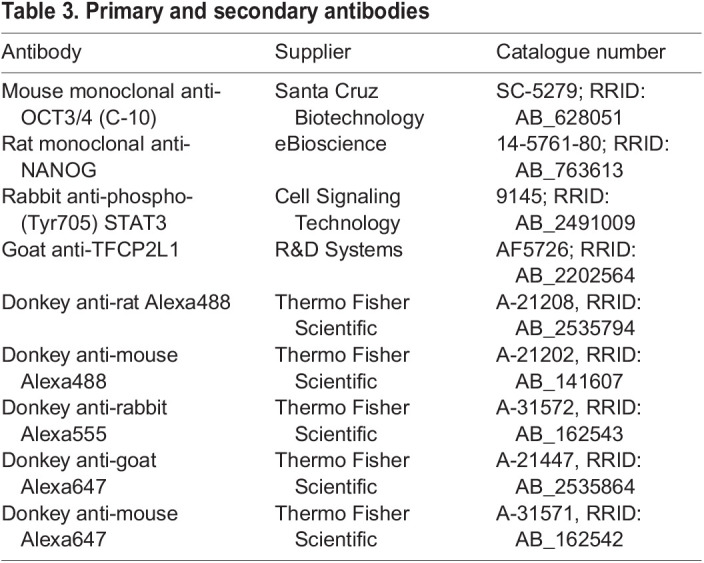
Primary and secondary antibodies

### Imaging and image analysis of embryos

Images for embryos were acquired using a TCS SP5 (Leica) confocal microscope and processed with ImageJ. All images have 8-bit depth, 1024×1024 pixels, 2 µm optical section thickness and were acquired on the ×20 (air) objective. Laser settings including exposure time and gain were kept consistent across experiments. However, it is not possible to prevent some quenching of fluorescence during image acquisition. Quantification of IF images was achieved using a computational pipeline to extract data on the intensity of antibody staining in individual nuclei. The 2D StarDist segmentation Fiji plugin (parameters: ‘percentileBottom’:‘1.0’, ‘percentileTop’:‘99.9’, ‘probThresh’:‘0.2’, ‘nmsThresh’:‘0.2’) was used to segment the DNA channel of IF images before using the Trackmate plugin to ‘track’ objects through the *z*-stack to generate 3D objects (parameters: area threshold>5, <30, track duration>5) (Ershov et al., 2022). An unsharp mask was applied to the DNA channel prior to segmentation (parameters: radius, 15; mask, 0.6). Nuclear segmentation of embryos was used as a mask to measure fluorescence intensity, xyz position and morphological parameters (e.g. volume) of each nucleus (Ollion et al., 2013; Pietzsch et al., 2015). Assessment of the histograms for signal intensity in the DNA channel and nuclear volume allowed thresholds to be set to remove erroneously segmented nuclei (upper limit for mean DAPI signal, 10; lower limit for mean DAPI signal, 2; upper limit for volume, 700; lower limit for volume, 150). Violin plots were generated using the ggplot2 package in R. To assess whether the difference in expression intensities between the embryonic stages (E3.5, E4.5 and diapaused) was statistically significant, a non-parametric, pairwise, unpaired Wilcoxon rank sum test was carried out within the R function ‘stat_compare_means’ and plotted over the violin plots. The non-parametric Wilcoxon rank sum test assumes the data are formed of one categorical, independent variable and one continuous dependent variable and that the observations are independent (e.g. no nucleus is in more than one group). As the variance between the groups was not equal (assessed with a Levene's test) nor was the data normally distributed (assessed with a Shapiro–Wilk test), the Wilcoxon rank sum test null hypothesis was that the distributions of the data across the E3.5, E4.5 and diapaused embryos was the same. The Wilcoxon rank sum test is robust to unequal group sizes, allowing comparisons across the embryo stages despite differences in the number of nuclei quantified. Additionally, a Kruskal–Wallis test was performed to assess whether the data followed the same distribution in embryos of different stages without the assumption of normality. *P*≤0.05 was considered significant.

### Generation of ‘*in silico*’ embryos

When objects (putative nuclei) identified by the Stardist-Trackmate pipeline are exported from Fiji, their xyz centroid coordinates, as well as the bounding box of the object are recorded. The exported csv file contains a single line of data for each object with the xyz centroid, shape parameters (e.g. volume) and fluorescence intensity parameters, including the average expression of a channel within every pixel of the object and sums of the expression values of every pixel in the object. Using the R function plotly, objects can be plotted into 3D space based on their xyz centroid coordinates and then colour coded according to the desired parameter, e.g. pSTAT3 intensity. Nuclei were visualised in a 2D gene expression space based on NANOG and STAT3 intensity, and selected nuclei were plotted back into the ‘*in silico*’ embryos, based on their positional information, and colour coded by STAT3 expression levels.
